# Explicating gender disparity in wearing face masks during the COVID-19 pandemic

**DOI:** 10.1186/s12889-022-14630-7

**Published:** 2022-12-05

**Authors:** Kim Hoe Looi

**Affiliations:** Xiamen University Malaysia, Bandar Sunsuria, Malaysia

**Keywords:** COVID-19 pandemic, Wearing face masks, Personal hygiene, Gender disparity in wearing face masks, Gender-based public health messages

## Abstract

**Background:**

The available evidence suggests that women were more likely to wear face masks as a precaution during the COVID-19 pandemic. However, few studies have explicated this gender disparity in wearing face masks. This study investigates associations of demographic factors with wearing face masks in Malaysia during the COVID-19 pandemic, then explicates gender disparity in wearing face masks from the lens of the Protection Motivation Theory.

**Methods:**

The first part of this study employed a structured online survey of 708 Malaysian adult participants. Data collected were quantitatively analyzed by means of descriptive statistics, bivariate correlations, analysis of variance (ANOVA), and multiple linear regression. The second part of this study was conducted among 28 women to better understand gender disparity in protection motivations from the perspectives of women.

**Results:**

Gender has the strongest positive association with wearing face masks (*p-*value < .001), followed by age (*p-*value = .028). The Protection Motivation Theory adequately explicated the gender disparity in wearing face masks. Additionally, women were motivated to wear face masks beyond protection from the SARS-CoV-2.

**Conclusion:**

Understanding the underlying motivations for wearing face masks informs design of gender-based public health messages to increase compliance with public health regulations and reduce morbidity and mortality for present and future public health crises.

**Supplementary Information:**

The online version contains supplementary material available at 10.1186/s12889-022-14630-7.

## Background

Despite the availability of vaccines since December 2020, the Coronavirus disease 2019 (COVID-19) remains unabetted due to new waves and new variants of the severe acute respiratory syndrome coronavirus 2 (SARS-CoV-2). Moreover, there are other issues related to vaccines, such as supply constraints [1], cost of procurement, unequitable distribution, stockpiling for strategic reasons, concerns about the effectiveness of vaccines against new variants of SARS-CoV-2, side effects from vaccinations, waning vaccine effectiveness over time [2, 3] and the well-described phenomenon of breakthrough infection after vaccination [2, 4].

Although a large section of the population was vaccinated, wearing face masks in specific situations and public settings remained an important public health intervention to protect healthy persons (i.e., prevention) and suppress onward transmission of SARS-CoV-2 from one infected individual to the others (i.e., source control) [5–8]. Because face masks offer protection against all variants of SARS-CoV-2, the Centers for Disease Control and Prevention [9] recommends that fully vaccinated people wear a face mask in public settings irrespective of the level of community transmission.

However, wearing a face mask per se is insufficient to prevent the spread of SARS-CoV-2 [7, 10] and may create a false sense of safety [7, 11, 12]. Consequently, at the individual level, a combination of nonpharmaceutical interventions are essential to reduce the transmission of SARS-CoV-2, such as the usage of personal protective equipment (PPE), improved personal hygiene, improved ventilation, reduction of attendance at gatherings, and physical barriers or physical distancing, is essential [13, 14]. The World Health Organization recommends using PPE, such as hand gloves, hand sanitizer, face shields, and medical/surgical face masks, as part of a comprehensive strategy for infection prevention and control of the transmission of SARS-CoV-2 [7, 15].

Greenhalgh et al. [11] contend that absence of evidence is not evidence of absence. Using the principle of precaution, the authors suggest that there is little to lose and potentially something to gain from wearing face masks. Wearing face masks as a precaution has been escalated to double masking, that is, wearing a cloth mask over a surgical mask [16]. Wearing a face mask provides various benefits as masks are cheap and easy to use, have strong sustainability, and are good for health and economy [6, 10–12, 17, 18], in contrast to other stringent measures (for instance, isolation and social-distancing) with significant societal and economic costs [12, 19]. The extant body of literature suggests that wearing face masks is contingent upon various factors. Fundamental factors include feasibility of use, availability, affordability, and comfort [7, 20]. Contextual factors include demographics, individual values, culture, and social norms [7, 18, 21].

Moreover, the effectiveness of face masks to prevent and control the transmission of SARS-CoV-2 and its variants is contingent upon correct wearing of face masks and community-wide adoption most of the time [5–7, 10–12, 18, 20, 22]. Modeling results suggest a potentially high value of wearing face masks by the general public to curtail community transmission of SARS-CoV-2 [6]. Community-wide wearing face masks is not a new nonpharmaceutical intervention. Dr. Lien Teh Wu, a Malayan epidemiologist, pioneered the development of surgical face masks and encouraged medical staff and the public to wear them during the Manchurian plague of 1910–1911 [23]. However, individual compliance for wearing face masks varies [8, 24, 25]. For instance, although wearing face masks in Malaysia was mandatory from August 1, 2020, the highest share for wearing face masks was 92% [26].

Prior research suggests that, in general, women are more risk averse than men [27], including the health/safety domain [28, 29]. Women were generally much more likely than men to appraise threat from SARS-CoV-2 as a severe health problem while men tend to have a more cavalier and macho attitude [28]. Men were more optimistic that they would not be seriously affected by the COVID-19 pandemic [30], although men were generally more susceptible to infectious diseases than women [31, 32]. Recent evidence suggests that the morbidity and mortality related to the COVID-19 pandemic are higher in men than in women [33–35]. This worldwide phenomenon, with few exceptions, is supported by a meta-analysis [36]. The available evidence suggests a gender divide in personal hygiene, with women being better at proper personal hygiene than men [33, 37–39]. Additionally, women were more likely to wear face masks as a precaution during the COVID-19 pandemic [30, 40] and the Severe Acute Respiratory Syndrome (SARS) epidemic [41]. Men were more likely to perceive wearing face masks as shameful, uncool, a sign of weakness, a stigma, threatening their masculine image and infringing their independence [30, 38, 42–45]. In sum, men are the “weak links in hygienic discipline” [46].

The public health response to the COVID-19 pandemic requires large-scale and significant shifts in individual behavior [18, 28] and imposes significant psychological burdens on individuals. As such, insights from the discipline of psychology can help align human behavior with recommendations of epidemiologists and public health experts [18]. The Protection Motivation Theory [47] postulates that increases in threat severity, threat vulnerability, response efficacy, and self-efficacy promote adaptive behaviors to protect oneself or others. Antithetically, decreases in maladaptive response rewards and adaptive response costs promote adaptive behaviors to protect oneself or others. Response efficacy is the belief that adaptive behavior will be effective in protecting oneself or others. Self-efficacy is the perceived ability to perform adaptive behavior. Response costs are costs related to engaging in adaptive behavior, such as monetary, time, effort, etc.

An understanding of the underlying motivations of wearing face masks is critical for targeted messaging to increase the overall prevalence of wearing face masks among groups with lower rates of wearing face masks [40] during public health crises. As such, this study investigates associations of demographic factors with wearing face masks in Malaysia during the COVID-19 pandemic and explicates gender disparity in wearing face masks from the lens of the Protection Motivation Theory.

## Methods

### Study design

The research methodology for the first part of this study was quantitative using a survey to collect data. The time frame of this study was cross-sectional.

### Measures

This self-administered online questionnaire contained demographic variables [13], such as gender (coded as 1 = man and 2 = woman), ethnicity, occupation, age, numbers of children under 18 years old living in the same household, health condition [13] (ranging from 1 = poor, 2 = fair, 3 = good, 4 = very good, and 5 = excellent), frequency of washing hands (ranging from 1 = never to 7 = every time), sufficient personal protective equipment (ranging from 1 = never to 5 = always), and frequency of wearing face masks (ranging from 1 = never to 7 = every time) [24, 43]. The questionnaire used for this study is in the Additional file 1.

To minimize response and measurement biases, this study followed the standard survey approaches [48], such that there was no social pressure to influence responses, no questions that would provoke defensiveness or threaten esteem, and no payoff or cost for particular responses. Given the multicultural nature of Malaysia, the questionnaire is offered in three major languages: Malay, Mandarin, and English [49].

### Participants

Malaysian adults above the age of 18 years old were eligible to participate in this survey (i.e., inclusion criteria). Participation in this survey was voluntary, and participants could opt out at any time. Moreover, participants were assured of the anonymity and confidentiality of their responses. All participants consented to participate in this online survey. For the second part of this study, 28 women provided their views on gender disparity in wearing face masks.

### Data collection

This study used Google Forms, an online survey questionnaire, which was a safe and feasible way of collecting data during the pandemic [50]. This study utilised several data collection strategies, including professional and personal networks [51] and snowball sampling technique [52] to reach as many participants as possible all over the country. The links to access the online questionnaires were distributed via WhatsApp, Facebook Messenger and, email in May 2020. A valid response implies that the participant is a Malaysian, 18 years old or above, and answered all questions in the questionnaire. A total of 708 valid responses were received.

To better understand gender disparity in protection motivations from the perspective of women, the second part of this study was conducted among 28 women. The open-ended question mentioned, “I conducted a survey recently and found that women were more likely to put on face masks than men. The existing literature suggests that men perceive wearing face masks as incongruent with their masculine image. In other words, wearing face masks infringes on men’s independence or men wearing face masks may be perceived by others as weak. However, I would like to explain this phenomenon from women’s perspective: why women are more likely to wear face masks than men. As such, I would like to solicit your personal opinions. Please feel free to express your opinions as there are no right or wrong answers. The results will be presented in aggregate, and no person will be identified.”

### Data analysis

Data collected from the online questionnaire were analyzed using the Statistical Package for Social Sciences software (SPSS), version 26. Descriptive statistics were used to analyze participants’ demographics. The analysis of variance (ANOVA) checked gender disparities in health condition, usage of PPE, frequency of washing hands and frequency of wearing face masks. The multiple linear regression checked the nature and degree of association between the independent variables of demographics and the dependent variable of the frequency of wearing face masks. Data collected from the second part of this study were analyzed for their contents.

## Results

The data were checked for normality using the normal Q–Q plot and results suggested that there was no serious violation. Thus, the use of parametric tests such as the ANOVA and the multiple linear regression was justified. Table 1 shows the descriptive statistics of the participants. The survey received responses from all the states and federal territories in Malaysia. The percentage of men and women participants was almost equal. Moreover, the participants were also found to be heterogenous. In contrast to Cowling et al. [17], who reported a lower level of wearing face masks compared with hand hygiene and other nonpharmaceutical interventions, this study found a higher level of wearing face masks compared with washing hands and usage of PPE. This result could be due to wearing face masks is visible. Hence, there were higher levels of wearing face masks in Malaysia.

**Table 1:** Demographic and descriptive statistics of participants

Variable	Frequency (%)	Mean (Standard deviation)
**Gender**		
Men	345 (49%)	–
Women	363 (51%)	
**Ethnicity**		
Malay	348 (49.2%)	–
Chinese	234 (33.1%)	
Indian	41 (5.8%)	
Natives of East Malaysia	85 (12.0%)	
**Occupation**		
Self-employed	80 (11%)	–
Employee	582 (82%)	
Student	4 (1%)	
Unemployed	42 (6%)	
**Age bracket**		
18 to 29 years old	101 (14%)	41.7 (11.1)
30 to 39 years old	213 (30%)	
40 to 49 years old	200 (28%)	
50 to 59 years old	159 (23%)	
60 years-old or above	35 (5%)	
**Numbers of children in the household**		
0	335 (46%)	2.2 (1.4)
1	119 (17%)	
2	112 (16%)	
3	83 (12%)	
4	41 (6%)	
5	14 (2%)	
6 or more	4 (1%)	
**Frequency of washing hands**		
Never	3 (1%)	5.3 (1.4)
Rarely (less than 10% of the time)	17 (2%)	
Occasionally (about 30% of the time)	60 (8%)	
Sometimes (about 50% of the time)	106 (15%)	
Frequently (about 70% of the time)	199 (28%)	
Usually (about 90% of the time)	119 (17%)	
Every time	204 (29%)	
**Usage of PPE**		
Never	7 (1%)	4.5 (.8)
Rarely	11 (2%)	
Sometimes	57 (8%)	
Often	170 (24%)	
Always	463 (65%)	
**Frequency of wearing face masks**		
Never	4 (1%)	6.3 (1.2)
Rarely (less than 10% of the time)	8 (1%)	
Occasionally (about 30% of the time)	15 (2%)	
Sometimes (about 50% of the time)	27 (4%)	
Frequently (about 70% of the time)	83 (12%)	
Usually (about 90% of the time)	104 (15%)	
Every time	467 (65%)	

The bivariate correlations (Table 2) revealed that gender (coded as 1 = man and 2 = woman) was positively correlated with the frequency of washing hands, usage of PPE, and frequency of wearing face masks. The frequency of washing hands and usage of PPE were correlated with the frequency of wearing face masks. Overall, the bivariate correlation results indicated the absence of multicollinearity.

**Table 2:** Bivariate correlations

	1	2	3	4	5	6
1. Gender	1					
2. Age	−.058	1				
3. Numbers of children	−.103^**^	.127^**^	1			
4. Frequency of washing hands	.086^*^	.221^**^	.051	1		
5. Usage of PPE	.078^*^	.062	.074^*^	.320^**^	1	
6. Frequency of wearing face masks	.126^**^	.067	−.011	.291^**^	.449^**^	1

Notes: * Correlation is significant at the 0.05 level (2-tailed). ** Correlation is significant at the 0.01 level (2-tailed)

Table 3 summarizes the analysis of variance (ANOVA) results for health condition, usage of PPE, frequency of washing hands, and frequency of wearing face masks between men and women. There were statistically significant differences in these variables between men and women. This finding corroborates prior studies that women consistently have a higher rate of wearing face masks than men [53–55].

**Table 3:** Summary of ANOVA

Variable		N	Mean	Standard deviation	F	*p-*value
Health condition	Men	345	3.7	.9	9.198	.003
	Women	363	3.5	.9		
	Total	708	3.6	.9		
Frequency of washing hands	Men	345	5.2	1.5	5.297	.022
	Women	363	5.5	1.3		
	Total	708	5.3	1.4		
Usage of PPE	Men	345	4.5	.8	4.363	.037
	Women	363	4.6	.7		
	Total	708	4.5	.8		
Frequency of wearing face masks	Men	345	6.2	1.2	11.408	.001
	Women	363	6.5	1.0		
	Total	708	6.3	1.2		

Table 4 presents the multiple linear regression results using the dependent variable of the frequency of wearing face masks. Among the demographic factors, gender (coded as 1 = man and 2 = woman) was the strongest positive predictor of the frequency of wearing face masks (*p-*value < .001), followed by age (*p-*value = .028). All variance inflation factors (VIFs) were close to one, indicating the absence of multicollinearity. Taken together, the results from Tables 3 and 4 indicated that women were more likely to wear face masks than men.

**Table 4:** Demographic factors associated with the frequency of wearing face masks

Variable	Standardized coefficients (Beta)	t	*p-*value	Collinearity Statistics	
				Tolerance	VIF
Gender (coded as 1 = man and 2 = woman)	.136	3.590	.000	.963	1.038
Age	.083	2.203	.028	.966	1.036
Ethnicity	.015	.400	.690	.968	1.034
Occupation	.009	.230	.818	.993	1.007
Numbers of children	−.006	−.150	.881	.925	1.081
Health condition	.070	1.850	.065	.971	1.030

To better understand gender disparity in protection motivations from women’s perspectives, that is, alternative explications to masculinity found in the extant literature, the second part of this study was conducted among women. The responses from 28 women were then classified by the frequency of each element in the Protection Motivation Theory (Table 5) and are described below.

**Table 5:** Frequency of each element in the Protection Motivation Theory

Appraisal	Element	Frequency
Threat appraisal	Intrinsic rewards	6
	Extrinsic rewards	9
	Vulnerability	25
Coping appraisal	Response efficacy	7
	Self-efficacy	23
	Response costs	17

### Intrinsic rewards

Intrinsic rewards for women wearing face masks include boosting their confidence, having a sense of security, or reducing inferiority complexes. Wearing face masks can hide one’s social phobia or genuine facial expression without faking a smile. On the contrary, the intrinsic reward for men not wearing face masks is probably a sense of independence.

### Extrinsic rewards

Extrinsic rewards for women wearing face masks include covering blemishes on their faces and avoiding unwanted attention from men. Additionally, face masks can be a fashion or accessory item, for instance, face masks with different colors and designs. Men tend to perceive negative extrinsic rewards for wearing face masks. Interestingly, women disagree with men’s perception of threat to their masculine image because of wearing face masks, indicating that this perception may be a fallacy among men. Stated differently, men are prisoners of their own device.

### Vulnerability

Consistent with the recent literature [30], the women surveyed reported that men tend to believe that they will be relatively unaffected by the SARS-CoV-2. In other words, men underestimated health risks. In the first part of this study, men reported significantly better health conditions than women (*p-*value = .003 in Table 3). Many women mentioned that they were vulnerable (25 mentions), citing that they were more worried, anxious, cautious, careful, safety-conscious, and willing to take precautionary measures for safety. As such, women choose a safer and more comprehensive approach, motivating them to be more alert about their health and practice better personal hygiene to prevent infection by the SARS-CoV-2, which explains why women reported higher levels of PPE usage, a higher frequency of washing hands and a higher frequency of wearing face masks in the first part of this study (Table 3).

### Response efficacy

The perceived weaker immune system among women may lead to their greater response efficacy, that is, they believe that the adaptive behavior of health and personal hygiene will be effective in protecting themselves and others against infection by the SARS-CoV-2. This response efficacy is also manifested in women’s self-efficacy.

### Self-efficacy

Women’s self-efficacy (i.e., perceived ability) was divided into compliance and social responsibility/altruism. Many women mentioned that women were naturally more obedient, compliant, and strictly abided by the law to wear face masks. Many women felt more responsible/altruistic toward their family and community. Their “mother’s instinct” coupled with their altruistic values (14 mentions) motivated them to protect themselves first, to love, care and protect people around them, such as reminding the whole family to wear face masks. These findings corroborated with a previous study [28]. In sum, it can be deduced that women have a high coping ability for the COVID-19 pandemic.

### Response costs

Women’s response costs for wearing face masks were reduced in terms of saving their time (i.e., less preparation) and lower expenses for make-up (17 mentions). Physiologically, women considered wearing face masks as another thing that they need to wear with a small marginal cost, whereas men’s response costs were higher because they perceived wearing face masks as an additional preparation or something troublesome.

## Discussion

### Principal findings

Consistent with a recent study [42], this study found that women reported significantly higher levels of usage of PPE, frequency of washing hands and frequency of wearing face masks than men (Table 3). The triangulation of findings from this study with previous study provided scientific evidence in support of the notion that men were the “weak links in hygienic discipline” [46].

Prior studies consistently found that women were more likely to wear face masks than men but failed to offer any coherent explanation. The literature suggests several explanations as to why men have higher rates of morbidity and mortality associated with the COVID-19 pandemic than women, broadly known as gender differences in biological, psychological, behavioral, and social factors [37]. Biologically, women have stronger immune responses against the SARS-CoV-2 [34, 56]. Behaviorally, men tend to engage in high-risk behaviors or lifestyles, leading to higher morbidity and mortality as a consequence of the COVID-19 pandemic [33, 34, 37, 56].

Scholars have called for public health and health-promotion interventions for changing a variety of health behaviors to be based on social and behavioral science theories [57]. In response to this call, this study explicated gender disparity in wearing face masks from the theoretical lens of the Protection Motivation Theory [47] supported by empirical evidence. This approach of health behavior theory in perspective [57] revealed a variety of underlying psychological motivations why women were more likely to wear face masks than men.

In contrast to Teasdale et al.’s [58] findings that coping appraisal solely predicted behavioral responses to pandemic flu, the present study employed the threat appraisal and coping appraisal of the Protection Motivation Theory to more comprehensively explain the gender divide in protection motivations for the COVID-19 pandemic. In terms of threat appraisal, vulnerability was most frequently cited by women. In terms of coping appraisal, women perceived response efficacy (i.e., effectiveness of their responses to threat), self-efficacy (especially their altruism), and negative response costs (e.g., wearing a face mask lower their costs compared with make-up), resulting in their high coping appraisal. In sum, the combination of threat appraisal and coping appraisal increased protection motivations of women in comparison with men, as predicted by the Protection Motivation Theory. Additionally, women were motivated to wear face masks beyond protection from the SARS-CoV-2. Their motivations can include cosmetic, fashion, social and reduced response costs unexplored by prior studies.

Understanding the cognitive beliefs (i.e., motivations) of wearing face masks underlying gender difference, it may be possible to change these beliefs. There is sufficient evidence at this point to suggest that men need more convincing and/or incentive to wear face masks than women. Consequently, designing men-friendly public health messages is a mainstay in the twenty-first century [38]. To encourage safer behaviors and reduce the spread of the disease, public health officials can consider the insights generated from this study, which go beyond prior suggestions to superficially target pandemic messaging to men differently than to women. Based on the responses from the women surveyed in the part two of this study, it appeared that multiple strategies may be necessary to increase the frequency of wearing face masks by men. Public health messages to promote wearing face masks directed toward men should highlight the illusions of invulnerability, promote men’s perceived and actual ability (i.e., self-efficacy) to engage in preventative behaviors when in public, anticipate and address men’s negative psychological beliefs about the likely costs (e.g., wearing a face mask is not unmanly, the fallacy of masculine image or producing macho face masks for men), feature men’s responsibilities to protect themselves and others (e.g., “You are our hero”), and emphasize the benefits of preventative behaviors to reduce the impacts of current and future public health crises. Given that men underestimated health risks, more information on health risks should be targeted at men to increase their compliance behaviors. Parenthetically, enlisting credible sources to appear in public health messages may be effective in shaping men’s preventative behaviors [18].

### Limitations and future work

This study has several limitations that can be addressed by future research. First, the sample is not representative. Thus, the results are not generalizable. A more representative sampling method is warranted to improve the generalizability of future findings. Second, in terms of methodology, this study used an online survey. Thus, people with no internet access, and lack of computing literacy not being surveyed. Third, the results might be subject to social desirability bias due to the self-report of participants. Fourth, other potential covariates (e.g., comorbidities) were not surveyed in the present study, which may positively bias the results. Fifth, in addition to combating epidemics or pandemics, there are many other issues related to wearing face masks, including social and cultural issues. Future research investigating factors associated with wearing face masks post the COVID-19 pandemic in different settings would be valuable in improving pandemic preparedness.

## Conclusion

Wearing face masks in public places has become a global symbol of the fight against the COVID-19 pandemic. The World Health Organization has warned that more lethal viruses will emerge in the future [59]. As such, it is envisaged that wearing face masks will continue to play an important role in infection prevention and control during the next pandemic until a new vaccine is widely available.

Explicating gender disparity in the frequency of wearing face masks is an interesting phenomenon worth exploring. This study found that being a woman has the strongest positive association with the frequency of wearing face masks and provided evidence that gender disparity can be adequately explicated by the Protection Motivation Theory. The central contribution of this study lies in offering a coherent explication for gender disparity in the frequency of wearing face masks via a psychological lens. Understanding the underlying motivations for wearing face masks informs design of gender-based public health messages to increase compliance with public health regulations and reduce morbidity and mortality for present and future public health crises.

## Supplementary Information

**Additional file 1. **Questionnaire.

## Data Availability

The data generated and/or analysed during the current study is available in the Mendeley Data repository, https://data.mendeley.com/datasets/hvg8wd6jdp/3.
