# Supplementary material for: Explicating gender disparity in wearing face masks during the COVID-19 pandemic
Source: BMC Public Health. 2022 Dec 5;22:2273. doi: 10.1186/s12889-022-14630-7 (PMC9724360; doi:10.1186/s12889-022-14630-7)
Supplement: Supplementary file 1 — Additional file 1. Questionnaire. [file 12889_2022_14630_MOESM1_ESM.docx]

**Questionnaire**

1. My gender:

1. Male
2. Female

2. My year of birth: _______ (for example, 1990)

3 . How many child/children under 18 years old is/are living together with you now?

a) None

b) One child

c) Two children

d) Three children

e) Four children

f) Five children

g) Six or more children

4. My ethnic group:

1. Malay
2. Chinese
3. Indian
4. Natives of Sabah or Sarawak
5. Others (please specify)______________

5. My occupation:

1. Self-employed
2. Employee
3. Student
4. Unemployed

6. Do you have sufficient personal protective equipment (for example, hand gloves, hand sanitiser or face mask) when you go out of your residence?

1. Never
2. Rarely
3. Sometimes
4. Often
5. Always

7. How frequent do you wash your hands for 20 seconds or longer after touching anything outside of your house?

a) Never

b) Rarely (less than 10% of the time)

c) Occasionally (about 30% of the time)

d) Sometimes (about 50% of the time)

e) Frequently (about 70% of the time)

f) Usually (about 90% of the time)

g) Every time

8. How frequent do you wear a face mask when you go outside of your house?

a) Never

b) Rarely (less than 10% of the time)

c) Occasionally (about 30% of the time)

d) Sometimes (about 50% of the time)

e) Frequently (about 70% of the time)

f) Usually (about 90% of the time)

g) Every time
